# Less is more—the best test for anastomotic leaks in rectal cancer patients prior to ileostomy reversal

**DOI:** 10.1007/s00384-021-03963-1

**Published:** 2021-07-12

**Authors:** Simon Lindner, Steffen Eitelbuss, Svetlana Hetjens, Joshua Gawlitza, Julia Hardt, Steffen Seyfried, Christian Galata, Christoph Reissfelder, Flavius Sandra-Petrescu, Florian Herrle

**Affiliations:** 1 Department of Surgery, Medical Faculty Mannheim, University Hospital Mannheim, University of Heidelberg, Mannheim, Germany; 2 Institute of Medical Statistics and Biomathematics, Medical Faculty Mannheim, University of Heidelberg, Mannheim, Germany; 3 Department of Radiology, Medical Faculty Mannheim, University Hospital Mannheim, University of Heidelberg, Mannheim, Germany

**Keywords:** Ileostomy reversal, Anastomotic leak, Diagnostic test accuracy, Endoscopy, Digital rectal examination, Contrast enema

## Abstract

**Purpose:**

No clear consensus exists on how to routinely assess the integrity of the colorectal anastomosis prior to ileostomy reversal. The objective of this study was to evaluate the accuracy of contrast enema, endoscopic procedures, and digital rectal examination in rectal cancer patients in this setting.

**Methods:**

A systematic literature search was performed. Studies assessing at least one index test for which a 2 × 2 table was calculable were included. Hierarchical summary receiver operating characteristic curves were calculated and used for test comparison. Paired data were used where parameters could not be calculated. Methodological quality was assessed with the QUADAS-2 tool.

**Results:**

Two prospective and 11 retrospective studies comprising 1903 patients were eligible for inclusion. Paired data analysis showed equal or better results for sensitivity and specificity of both endoscopic procedures and digital rectal examination compared to contrast enema. Subgroup analysis of contrast enema according to methodological quality revealed that studies with higher methodological quality reported poorer sensitivity for equal specificity and vice versa. No case was described where a contrast enema revealed an anastomotic leak that was overseen in digital rectal examination or endoscopic procedures.

**Conclusions:**

Endoscopy and digital rectal examination appear to be the best diagnostic tests to assess the integrity of the colorectal anastomosis prior to ileostomy reversal. Accuracy measures of contrast enema are overestimated by studies with lower methodological quality. Synopsis of existing evidence and risk–benefit considerations justifies omission of contrast enema in favor of endoscopic and clinical assessment.

**Trial registration:**

https://www.crd.york.ac.uk/prospero/display_record.php?ID=CRD42019107771

**Supplementary information:**

The online version contains supplementary material available at 10.1007/s00384-021-03963-1.

## Introduction

Anastomotic leak is a much-dreaded complication after coloanal or colorectal anastomosis. The most common indication for this type of anastomosis is anterior resection for mid to low rectal cancer. Since the most serious implications of anastomotic breakdown include inflammation abscess or sepsis, rectal resection is usually combined with concurrent proximal fecal diversion through formation of a temporary ileostomy. Although anastomotic leak cannot be prevented by this measure, this greatly mitigates the incidence and impact of a clinical leak that can thus oftentimes be treated without reintervention [1, 2].

Before restoration of gastrointestinal continuity, however, the anastomosis is usually assessed for asymptomatic leaks that could become clinically apparent after reversal. Contrast enema (CE), digital rectal examination (DRE), and flexible or rigid endoscopic procedures (EP) are the most common examination techniques for this indication. All index tests can cause significant discomfort and furthermore occupy time of a skilled medical professional. Furthermore, EP are carried out under conscious sedation in some centers, accompanied with risks of respiratory and hemodynamic depression, and the risk of perforation has to be considered. During CE, dilation due to contrast agent instillation can be painful and thus sometimes requires sedation as well, and the patient is exposed to radiation. To make the diagnostic process prior to ileostomy reversal as convenient for patients and cost-effective as possible, an evidence-based algorithm would be desirable.

The target condition is asymptomatic anastomotic defect in patients awaiting ileostomy reversal that would lead to complications if gastrointestinal continuity was restored. This definition was chosen to separate clinically relevant asymptomatic leaks from previously described leaks that may be found in imaging test but lead to no complications after reversal [3, 4]. Clinical anastomotic leaks that were already suspected because the patient showed corresponding symptoms were not the target condition of this review. A systematic review and meta-analysis of CE for assessment of anastomotic integrity was published in 2015 by Habib et al. [5], but it remained unclear whether routine CE provides additional information over clinical assessment alone. However, new studies on CE have since been published, potentially leading to more insight. Moreover, Habib et al. did not assess methodological quality of the included studies, potentially leading to overestimation or underestimation of the utility of CE.

Furthermore, to this date no review and meta-analysis has been done that included other diagnostic tools prior to ileostomy reversal. This systematic review aims at a direct comparison of CE, DRE, and EP in search of an evidence-based diagnostic algorithm prior to ileostomy reversal.

## Methods

### Protocol and registration

Preliminary research was started on October 1, 2018. Formal literature search was done until December 31, 2018. Following the Preferred Reporting Items for Systematic Reviews and Meta-Analyses (PRISMA 2009) [6], an a priori protocol for this review was constructed and registered on January 10, 2019 in the PROSPERO database, an international prospective register of systematic reviews. It is accessible online via https://www.crd.york.ac.uk/prospero/display_record.php?ID=CRD42019107771 under register number CRD42019107771.

### Eligibility criteria

Prospective and retrospective clinical trials of cross-sectional, cohort, or case-control design were considered for this diagnostic test accuracy review. No language restrictions were applied. The reviewed setting is the diagnostic process prior to ileostomy reversal, for detection of asymptomatic anastomotic leaks after mid to low rectal resection and formation of a temporary ileostomy for rectal cancer. Preliminary searches had shown a scarcity of studies solely comprised of rectal cancer patients. Thus, studies with mixed underlying pathologies were included if the majority of the cohort (> 50%) consisted of rectal cancer patients. Types of anastomoses are end-to-end, side-to-end, and colonic J-pouch. Eligible index tests for assessment of anastomotic integrity were antegrade or retrograde contrast enema, digital rectal examination, and endoscopic imaging such as flexible sigmoidoscopy or rigid proctoscopy. The target condition was asymptomatic anastomotic leak. Outcome after ileostomy reversal serves as reference standard, preferably supported by other factors such as laboratory findings or further testing. Changes in clinical management alone, such as a delay in the reversal of the ileostomy in a non-blinded clinical study, were considered unfavorable reference standards by the authors of this review, as those characteristics are heavily influenced by the results of the index tests and thus are prone to bias. Studies reporting only whether patients had ileostomy reversed, without postoperative follow-up on the outcome, were not deemed eligible for this review.

### Information sources

A search of the electronic databases MEDLINE, Cochrane Library, Google Scholar, clinicaltrials.gov, CINAHL, and Web of Science, as well as cross search of references of relevant articles, was performed by two authors (SL, SE) in cooperation with an institutional database researcher. Data from an additional primary study, recently conducted by authors of this review, was included prior to its publication [7].

### Search strategy

As an example, the search strategy used for PubMed (MEDLINE) is presented in Table 1. Advanced search options including synonyms, truncations, and combinations were used. Search results were checked for inclusion of all literature that was found in preliminary searches.

**Table 1:** Basic search strategy used for the PubMed (MEDLINE) Database

(stoma*[tiab] OR "Ileostomy"[Mesh] OR ileostom*[tiab])
AND
(reversal[tiab] OR closure[tiab] OR reanastomosis[tiab] OR takedown[tiab])
AND
("Enema"[Mesh] OR Enema*[tiab] OR Pouchogra*[tiab]
OR
"Colonoscopy"[Mesh] OR Coloscop*[tiab] OR Colonoscop*[tiab] OR Sigmoidoscop*[tiab] OR "Proctoscopy"[Mesh]
OR Proctoscop*[tiab]
OR
"Digital Rectal Examination"[Mesh] OR Digital rectal examination*[tiab])

### Study selection

Two review authors (SL and SE) independently assessed the search results for eligibility. After removal of duplicates, the results were screened by title and abstract. Eligible studies were then checked by full text for inclusion in the review and meta-analysis. Definitive inclusion was then decided by discussion (SL, SE, and FH).

### Data extraction

Two review authors (SL and SE) independently extracted data using a data extraction form, tailored to this review question. A third review author (FH) evaluated any discrepant judgments. Data extraction of true positive, false positive, false negative, and true negative values to generate a 2 × 2 discrepancy table was performed for each study.

### Statistical analysis

Comparison of the index tests by analyzing differences in their respective HSROC curve was the primary aim of this meta-analysis. Investigating the influence of methodological quality was the secondary aim. For the calculation of the HSROC model parameters, the bivariate model parameters and the confidence and prediction regions of the SAS macro MetaDAS (version 1.3), provided by the Cochrane Collaboration [8], were used. This analysis was performed with SAS software, release 9.4 (SAS Institute Inc., Cary, NC, USA). Where HSROC parameters could not be calculated, informal analysis of paired data was performed. For this, index test results of comparative studies are plotted in the same diagram and connected with a dotted line to aid visual interpretation. Further comments on the statistical analysis are provided as supplementary material [Addendum 1].

### Assessment of methodological quality

The risk of bias in individual studies was assessed using QUADAS-2 [9] by two review authors (SL and SE). The tool content was tailored to the question of this systematic review [Addendum 2]. Differences were resolved by discussion and consensus between the review authors. All signaling questions that were changed, added, or omitted are listed in Table 2. For subgroup analysis, number of domains marked with high risk were calculated for each study, and studies were accordingly split into two as equal as possible sized groups.

**Table 2:** Changes to QUADAS-2 signaling questions after review-specific tailoring

Domain	Omitted signaling question	Added signaling question
2 Index test(s)	Were the index test results interpreted without knowledge of the results of the reference standard?	Were the index test results interpreted without knowledge of the results of the reference standard or other index test(s)?
2 Index test(s)	If a threshold was used, was it pre-specified?	Was the index test interpreted by a senior consultant?
3 Reference standard	Were the reference standard results interpreted without knowledge of the results of the index test?	Was the reference standard a clinical outcome or were results interpreted without knowledge of the results of the index test?
2 Index test(s)		Did surgeons and radiologists cooperate in conceiving the study/interpreting results?
4 Patient flow and timing	Was there an appropriate interval between index test(s) and reference standard?	Was the time interval between index test(s) and reference standard ≤ 30 days?

## Results

The database searches produced 330 articles. Twenty-three articles had already been identified through preliminary searches, one of those were data prior to publication. After removal of duplicates and screening by title and abstract, 25 full-text articles were assessed for eligibility. Of those, 11 retrospective [4, 7, 10–18] and 2 prospective studies [19, 20] were included in the review. The 12 excluded articles [3, 21–31] and the reasons for their exclusion are listed in Table 3. Characteristics of the included studies are shown in Table 4. The 13 included studies comprised 1903 patients. A 2 × 2 discrepancy table could be calculated for 1818 contrast enemas in 13 studies, 852 endoscopic assessments in 4 studies, and 779 digital rectal examinations in 5 studies. The PRISMA 2009 [6] flow diagram and PRISMA 2018 checklist [32] are provided as supplementary material (Supplementary Fig. 1) and (Supplementary Table 1).

**Table 3:** Reasons for exclusion after full text assessment

Author	Year	Reason for exclusion
Karanjia	1994	No assessment of diagnostic tools; thus no eligible data available
Lim	2006	Setting does not fit review question; no eligible reference standard
Khair	2007	Not all patients received index test; no eligible reference standard
Karsten	2009	No eligible reference standard; number of false negatives not stated
Phillips	2010	No diagnostic study; no eligible index test
Palmisano	2011	Ileostomy only in selected patients; no eligible reference standard
Killeen	2013	No reference standard available; calculation of 2 × 2 table not possible
Reilly	2014	Insufficient reference standard
Dimitriou	2015	No false/true negatives available; calculation of 2 × 2 table not possible
Seo	2015	No false/true negatives available; calculation of 2 × 2 table not possible
Sherman	2017	Literature overview; no eligible data available
Climent	2018	Missing data; calculation of the 2 × 2 table not possible

**Table 4:** Characteristics of the individual studies

First author	Year	Authors ‘ specialty	Country	Period (months)	Design	Median age (years)	Female (%)	Rectal cancer (%)	Cohort* (n)	CE^A^ (n)	EP^A^ (n)	DR^A^ (n)
MacLeod	2004	Surg, Rad	Great Britain	36	Prospective	64	48	100	52	40	0	40
daSilva	2004	Surg	United States	113	Retrospective	67 *(mean)*	36	80	84	77	0	0
Tang	2005	Surg	Singapore	45	Prospective	62	40	85	195	179	0	180
Cowan	2005	*unknown*	New Zealand	96	Retrospective	71	34	83	59	33	0	0
Kalady	2008	*unknown*	United States	96	Retrospective	53 *(mean)*	40	57	211	211	211	211
Jeyarajah	2008	Surg	Great Britain	*Unknown*	Retrospective	68	31	100	38	38	0	0
Hong	2012	Surg	South Korea	72	Retrospective	60 *(mean)*	42	100	145	144	0	0
Nabi	2013	*unknown*	Australia	60	Retrospective	52	39	90	122	118	0	0
Larsson	2015	Surg	Sweden	36	Retrospective	65 *(mean)*	40	96	95	50	50	50
Shalabi	2016	Surg	Israel	96	Retrospective	62 *(mean)*	59	100	312	298	298	298
Goetz	2017	Rad, Surg	Germany	74	Retrospective	60	38	52	252	274	0	0
Katory	2017	Surg, Rad	Great Britain	84	Retrospective	66	22	100	45	63	0	0
Lindner	2020	Surg, Rad	Germany	156	Retrospective	62	34	100	293	293	293	0

*CE* contrast enema, *EP* endoscopic procedure, *DRE* digital rectal examination

^*^Study cohort as stated in the full text

^A^number of tests for which a 2 × 2 discrepancy table could be calculated

### Methodological quality of included studies

**Fig. 1:**
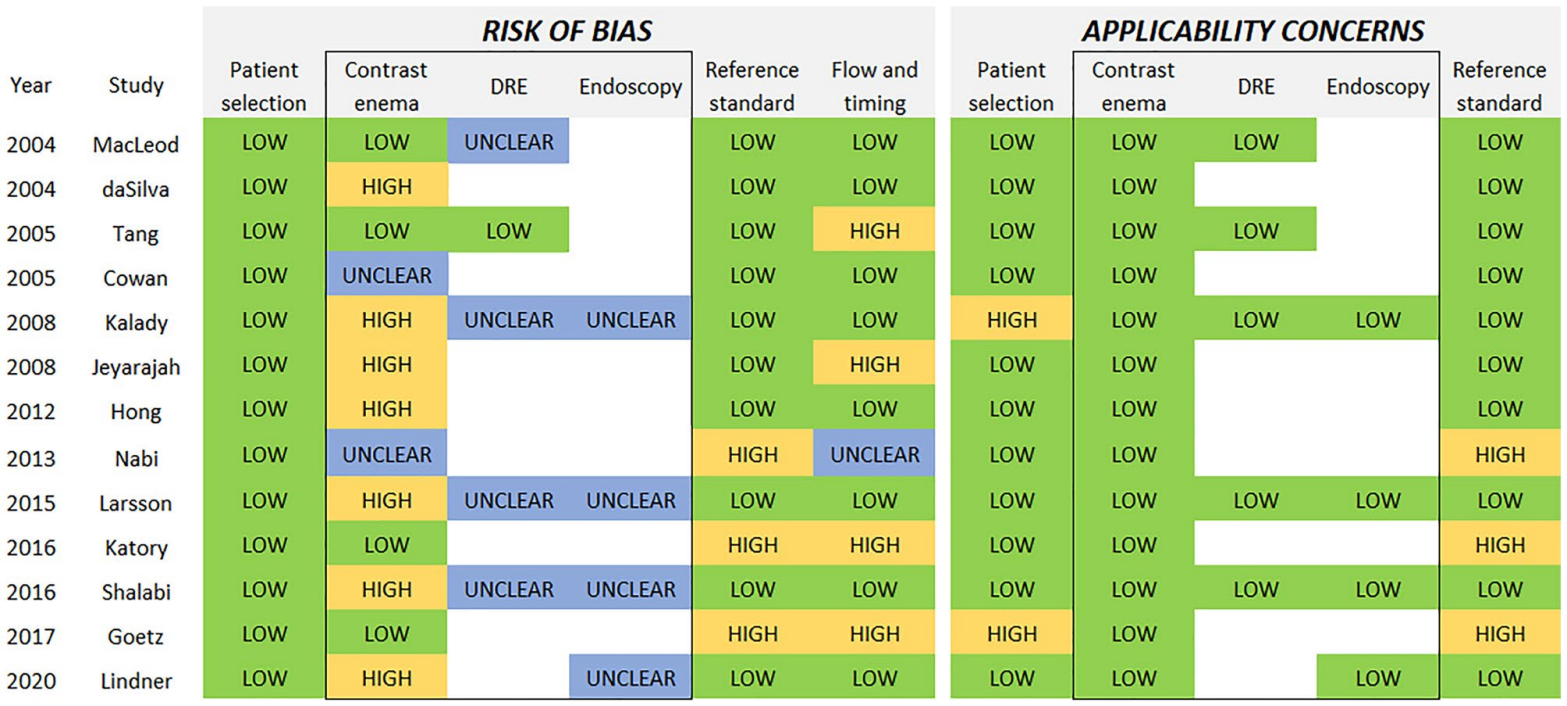
QUADAS-2 questionnaire results listed by individual study

Results of the QUADAS-2 assessment for each included study are shown in Fig. 1. The overall methodological quality of studies using CE as index test was poorer than that of studies also assessing DRE or EP.

### Proportion of underlying pathologies

As predicted by preliminary searches, there was a scarcity of studies that comprised solely of rectal cancer patients. Seven of the included 13 studies had patients with other underlying pathologies, such as other malignant dieseases, inflammatory bowel disease, or diverticulitis. However, of the 4 largest included studies with more than 200 patients each, 2 exclusively consisted of rectal cancer patients, and of the total 1903 patients in all included studies, 1619 (85%) were rectal cancer patients.

### Calculation of HSROC curves

**Fig. 2:**
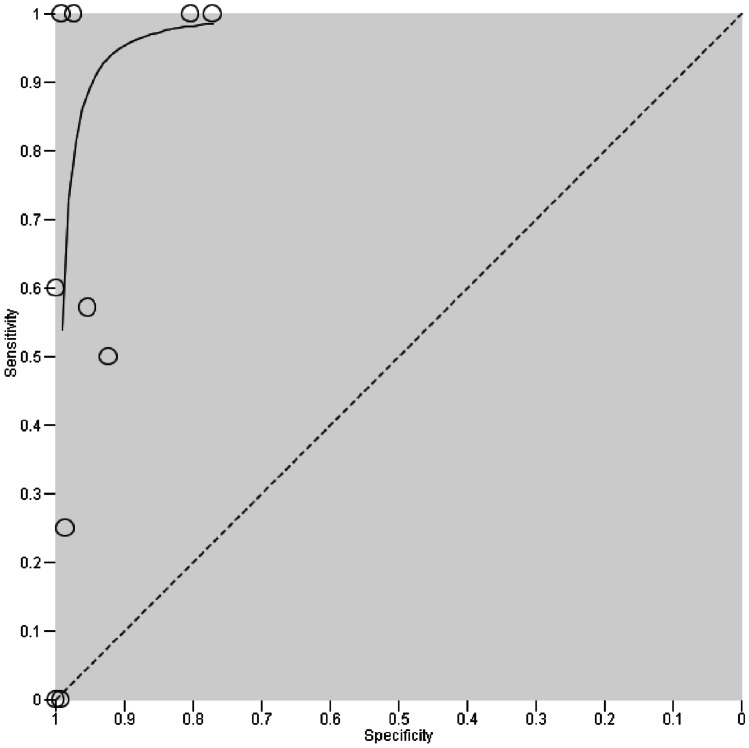
Overall diagnostic test accuracy of contrast enema. Each individual study is plotted by respective sensitivity and (1 − specificity). Test accuracy thus improves from bottom right corner to top left corner. As no fixed threshold can be assumed, a hierarchical summary receiver operating characteristic (HSROC) curve was calculated to summarize the findings. The HSROC curve represents the underlying correlation of sensitivity and specificity of all studies

For CE, an HSROC curve, representing sensitivity in relation to specificity, was calculated (Fig. 2). Too few studies were eligible to calculate HSROC parameters for EP and DRE.

### Diagnostic test accuracy of CE according to methodological quality

**Fig. 3:**
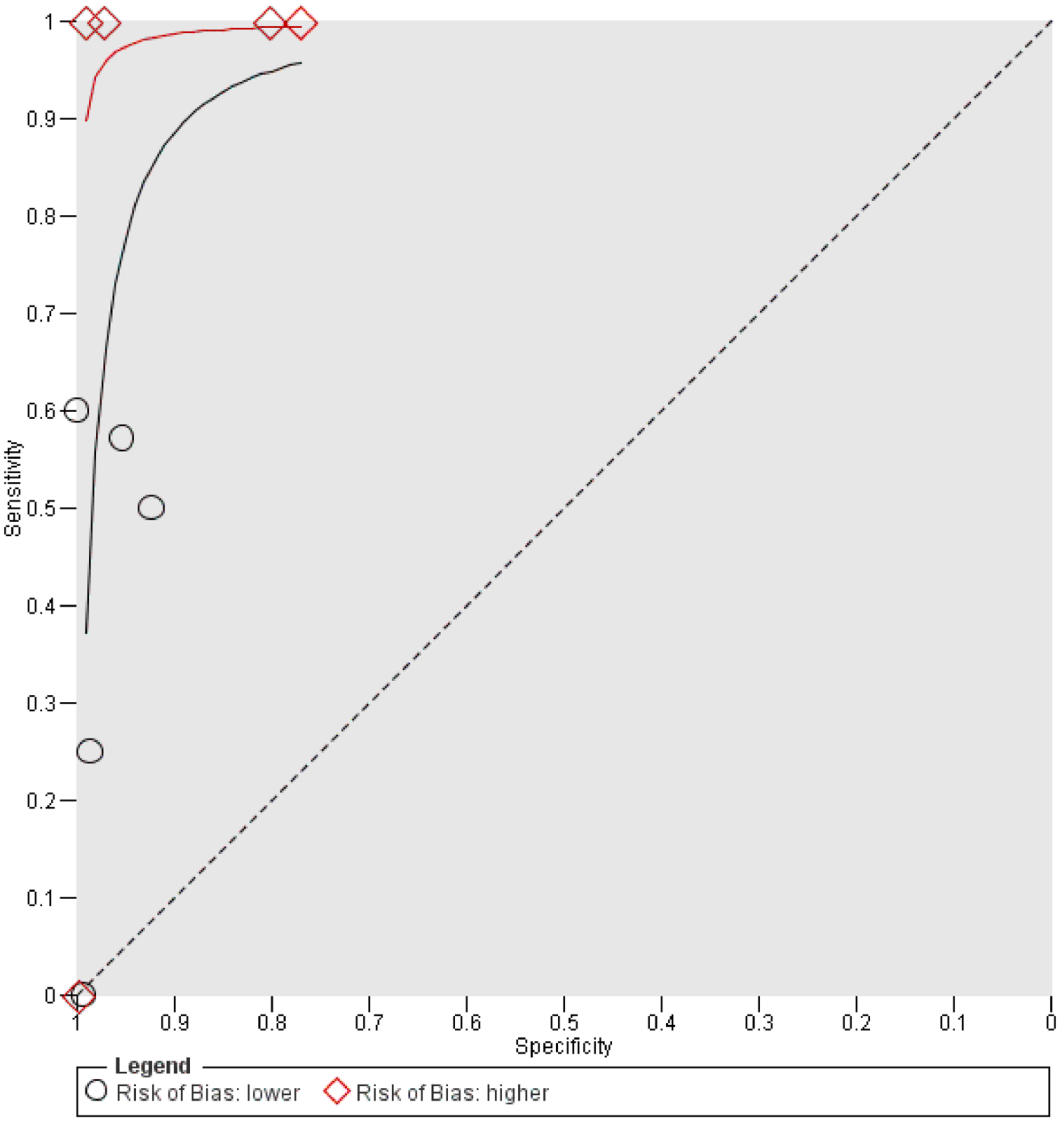
Subgroup analysis of contrast enema studies according to risk of bias. Each individual study is plotted by respective sensitivity and (1 − specificity). Test accuracy thus improves from bottom right corner to top left corner. For comparison according to risk of bias, the underlying diagnostic test accuracy of the groups with higher and lower risk of bias was assessed separately. As no fixed threshold can be assumed, a hierarchical summary receiver operating characteristic (HSROC) curve was calculated to summarize the findings for each group. The HSROC represents the underlying correlation of sensitivity and specificity of all studies in each group

There were 8 studies with one or less domains marked as high, thus comprising the lower risk of bias group, and 5 studies with two or more domains marked as high, thus comprising the higher risk of bias group. Subgroup analysis showed the overestimation of CE accuracy measures in the group with a higher risk of bias. There was higher sensitivity for equal specificity and vice versa (Fig. 3).

### Comparison of CE and EP

**Fig. 4:**
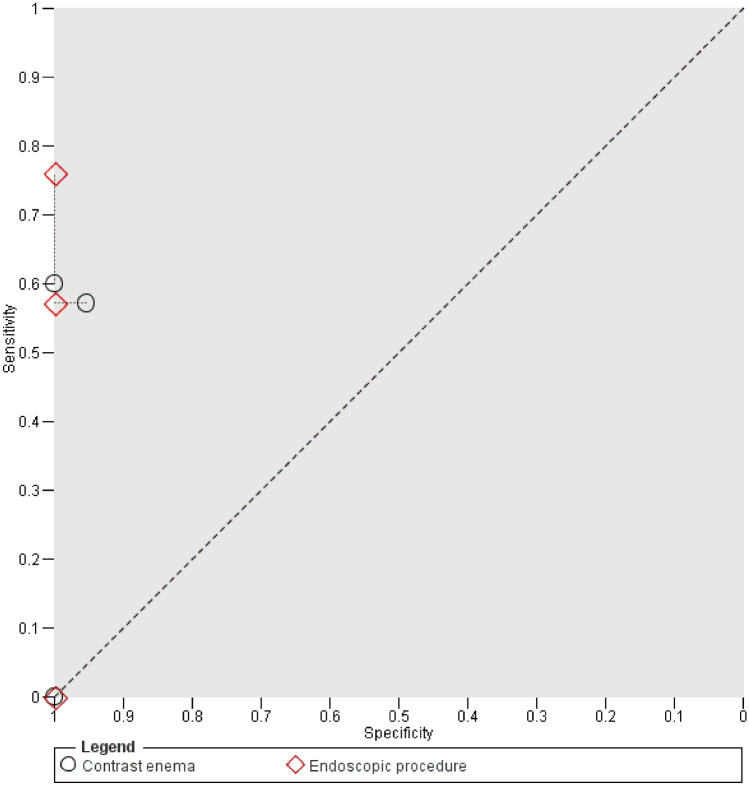
Paired data analysis of contrast enema and endoscopic procedures. Index test results of each comparative study are plotted by respective sensitivity and (1 − specificity). Test accuracy thus improves from bottom right corner to top left corner. Contrast enema and endoscopic results of each study are connected with a dotted line to aid visual interpretation

Since HSROC parameters for EP could not be calculated, informal analysis by paired data was performed (Fig. 4). This showed equal or superior accuracy measures of EP in all studies. There were 4 comparative studies [4, 7, 12, 16], but for one (Shalabi 2016) sensitivity could not be calculated due to a 0% incidence. Specificity of EP was superior to CE. In all assessed cases, no leak found by CE was overseen by EP.

### Comparison of CE and DRE

**Fig. 5:**
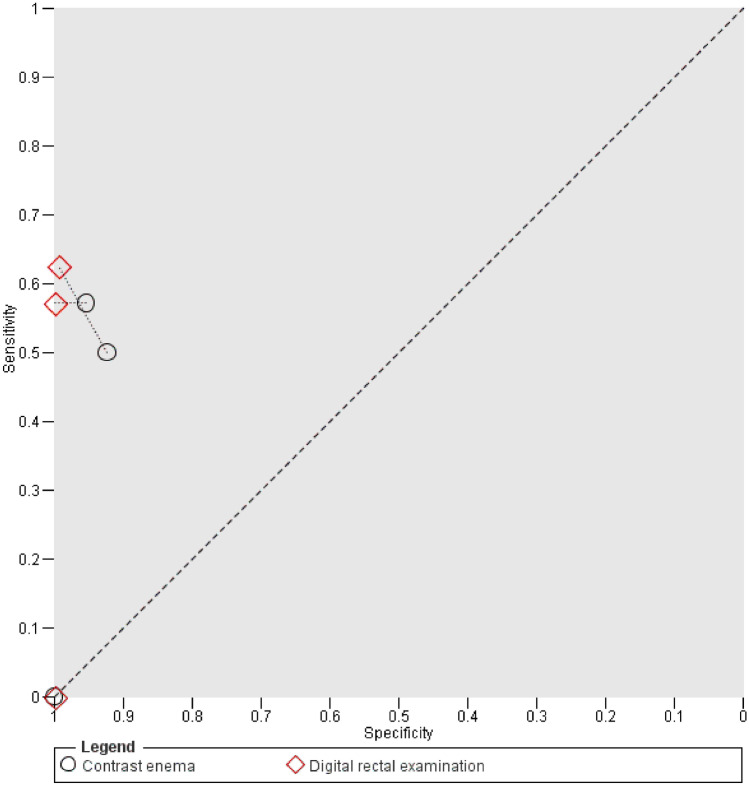
Paired data analysis of contrast enema and digital rectal examination. Index test results of comparative studies are plotted by respective sensitivity and (1 − specificity). Test accuracy thus improves from bottom right corner to top left corner. Contrast enema and digital rectal examination results of each study are connected with a dotted line to aid visual interpretation

HSROC parameters for DRE could also not be calculated; thus, again paired data were used to perform informal analysis (Fig. 5). In all studies, there were superior or equal accuracy measures of DRE compared to CE. For 2 (MacLeod 2004; Shalabi 2016) out of the 5 available studies [4, 12, 16, 19, 20] sensitivity could not be calculated due to a 0% incidence. In all assessed cases, no leak found by CE was overseen by DRE.

### Comparison of DRE and EP

**Fig. 6:**
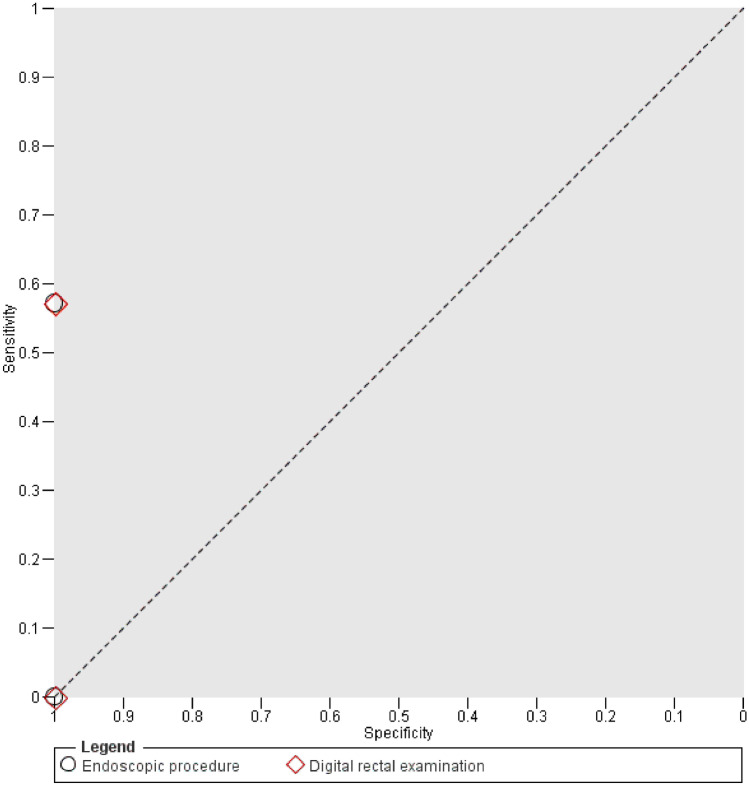
Paired data analysis of endoscopic procedures and digital rectal examination. Index test results of comparative studies are plotted by respective sensitivity and (1 − specificity). Test accuracy thus improves from bottom right corner to top left corner. Endoscopic and digital rectal examination results of each study are connected with a dotted line to aid visual interpretation

There were 3 studies available [4, 12, 16]. Sensitivity could not be calculated in one study due to a 0% incidence (Shalabi 2016). Paired data analysis of DRE and EP showed concordance of all findings and thus equal sensitivity and specificity (Fig. 6).

### Routine imaging vs. selective use

All diagnostic tools have unsatisfactory sensitivity in routine testing, and prevalence of asymptomatic anastomotic leakage is relatively low. To assess whether routine imaging of patients who had an uneventful postoperative course is useful, or if diagnostic tools should be reserved for those patients who developed anastomotic leakage after the initial operation, studies providing individual data on those two groups were assessed. There were only 3 available studies, with a total of 518 eligible patients without anastomotic leakage following rectal resection and 103 patients with previous anastomotic leak. Only one of those studies had intended a direct comparative assessment of these groups. Mean prevalence of asymptomatic anastomotic leak prior to ileostomy reversal in the group without previous leak was 0.5% (± 0.8%); thus, approximately 200 patients have to be tested to find one leak. In contrast, in the group that developed a leak after the initial operation, 16.4% (± 8.0%) showed an asymptomatic leak prior to ileostomy reversal. Here, only approximately six patients have to be tested in order to find an asymptomatic anastomotic leak.

## Discussion

This systematic review and meta-analysis demonstrates that sensitivity and specificity of CE for the detection of asymptomatic anastomotic leak are probably overestimated due to study bias. Studies with higher risk of bias reported better accuracy measures than those with lower risk of bias. Availability and tradition, rather than a supporting evidence, seems to be the explanation for the widespread use of CE throughout the world. There are too few studies assessing EP and DRE to make any definitive statements on the clinical value of those tests. Generally, they seem to be of at least comparable value. Studies including those tests were less prone to bias and paired data analysis indicates that both could be more accurate than CE. This is supported by the fact that no study reported a leak found in CE that was overseen by DRE or EP.

The acquired data suggest that routine use of any diagnostic tool prior to ileostomy reversal rarely detects significant anastomotic defects in patients that had no previously reported problems of their anastomoses. Omission of diagnostic tests in those patients has previously been proposed [28, 33]. Evidence is not strong, but the low prevalence in this group might support those views. In patients that had a leak after the initial operation, six have to be tested to find an asymptomatic leak. In patients without a previous leak this number rises to 200. However, this assessment was not the primary aim of this review, and prospective studies are needed to make any definitive judgments.

### Limitations of this review

This review and meta-analysis aimed at comparing the index tests after resection of rectal cancer. However, compromises had to be made to aquire sufficient data for statistical analysis. Overall, rectal cancer patients comprised 85% of included patients. Furthermore, there were too few studies assessing EP and DRE to fulfill the primary objective of the meta-analysis, a formal comparison of the index tests. Overall, methodological quality of the eligible studies was moderate and most studies were retrospective. Blinded prospective clinical trials are completely missing. The overall evidence on which this systematic review and meta-analyis is based is fragile and conclusions drawn from it must be well-considered. The primary aim, to deduct an evidence-based algorithm for the diagnostic process, could not be fulfilled.

### Implications for clinical practice

The collected evidence clearly points towards the omission of CE wherever an EP is available, since no additional information is gained. Of all 3 investigated tests it is also the one most associated with patient discomfort. Additionally, radiation exposure must be considered. There are limitiations to the evidence, as mentioned above. To gain stronger evidence, prospective and larger studies are needed. In our opinion, although desirable, it appears unlikely that those studies will be made. Thus, a pragmatic synopsis of existing evidence, patient comfort, and economic and risk-benefit considerations must be made. Another use for CE, the prediction of fecal incontinence, has been shown to be of little value [17, 34]. Although knowledge concerning diagnostic procedures for the detection of anastomotic strictures is very limited, in our experience this condition can be sufficiently assessed by EP, very low anastomoses even by DRE.

Since the use of CE is not supported by evidence, it may be omitted as a routine screening test for asymptomatic anastomotic leak prior to ileostomy reversal in rectal cancer patients when EP and DRE are available. 

## Conclusion

Endoscopy and digital rectal examination appear to be the best diagnostic tests to assess the integrity of the colorectal anastomosis prior to ileostomy reversal. Accuracy measures of contrast enema are overestimated by studies with lower methodological quality. Synopsis of existing evidence and risk–benefit considerations justifies omission of contrast enema in favor of endoscopic and clinical assessment.

## Supplementary information

Below is the link to the electronic supplementary material.

Supplementary file1 (DOCX 13 KB)

Supplementary file2 (DOCX 14 KB)

Supplementary file3 (TIF 2680 KB)

Supplementary file4 (DOC 67 KB)

## Data Availability

Data and methods used in the analysis are available from the corresponding author on reasonable request.
