# Supplementary material for: Less is more—the best test for anastomotic leaks in rectal cancer patients prior to ileostomy reversal
Source: Int J Colorectal Dis. 2021 Jul 12;36(11):2387–98. doi: 10.1007/s00384-021-03963-1 (PMC8505329; doi:10.1007/s00384-021-03963-1)
Supplement: Supplementary file 1 — Supplementary file1 (DOCX 13 KB) [file 384_2021_3963_MOESM1_ESM.docx]

**Comments on the statistical analysis**

All index tests of this meta-analysis have binary (leak yes/no) test results. Thresholds for positivity are discrete, non-numerical, due to possible inter-observer variability. Therefore, differences in sensitivity and specificity of two studies might only be attributable to different thresholds and could otherwise have produced the same results. Calculation of summary estimates of accuracy measures, however, is only possible in case of fixed thresholds. In this case, analysis of hierarchical summary receiver operating characteristic (HSROC) curves can be used for test comparison. HSROC diagrams plot each study with its sensitivity and specificity value. From the test results of each study, a ROC curve is calculated, representing the correlation of sensitivity and specificity. Comparison of the index tests by analyzing differences in their respective HSROC curve was thus the primary aim of this meta-analysis. Investigating the influence of methodological quality on the HSROC was the secondary aim.
