# Supplementary material for: Less is more—the best test for anastomotic leaks in rectal cancer patients prior to ileostomy reversal
Source: Int J Colorectal Dis. 2021 Jul 12;36(11):2387–98. doi: 10.1007/s00384-021-03963-1 (PMC8505329; doi:10.1007/s00384-021-03963-1)
Supplement: Supplementary file 2 — Supplementary file2 (DOCX 14 KB) [file 384_2021_3963_MOESM2_ESM.docx]

**Review-specific tailored QUADAS-2 items**

**Domain 2 - Index test(s).** Comparative studies are included in this study. To address the potential bias induced by non-blinded testing in comparative studies, the first signaling question of domain 2 was extended to assess blinding in respect to other performed index test(s).

Specification of a threshold cannot be accurately applied to the imaging tests subject to this review. In the authors’ opinion, the best guarantor of accurate interpretation of those imaging tests is the senior consultant, not only in cases where imaging tests were positive or unclear. Thus, the second item of domain 2 demands all images being reviewed by a senior consultant. This had to be explicitly stated in the study manuscript.

To assess reporting bias induced by the specialty of study authors, a third signaling question was added to domain 2. Surgeons and radiologists might have different opinions on the effectiveness of CE. When both specialties were involved in a study, the risk of reporting bias was deemed lower.

**Domain 3 - Reference standard.** For referencing anastomotic leak, some studies might use clinical outcomes such as leak requiring intervention after ileostomy reversal as a reference standard. Clinical outcomes are unlikely to be affected by bias. For positive findings, however, reversal of the ileostomy is an inappropriate reference standard. Here, other test results must be consulted. For the reference standard, using more than one other test for positive, and clinical outcomes for negative index test results was thus deemed to have the lowest risk for bias.

**Domain 4 - Patient flow and timing.** For the first signaling question, the appropriate time interval between index test(s) and reference standard was defined as 30 days or less. Longer time intervals might result in spontaneous healing of a subclinical or masked leak in between testing, and test results can thus not be accurately compared.
