# Supplementary figures and images for: Less is more—the best test for anastomotic leaks in rectal cancer patients prior to ileostomy reversal
Source: Int J Colorectal Dis. 2021 Jul 12;36(11):2387–98. doi: 10.1007/s00384-021-03963-1 (PMC8505329; doi:10.1007/s00384-021-03963-1)

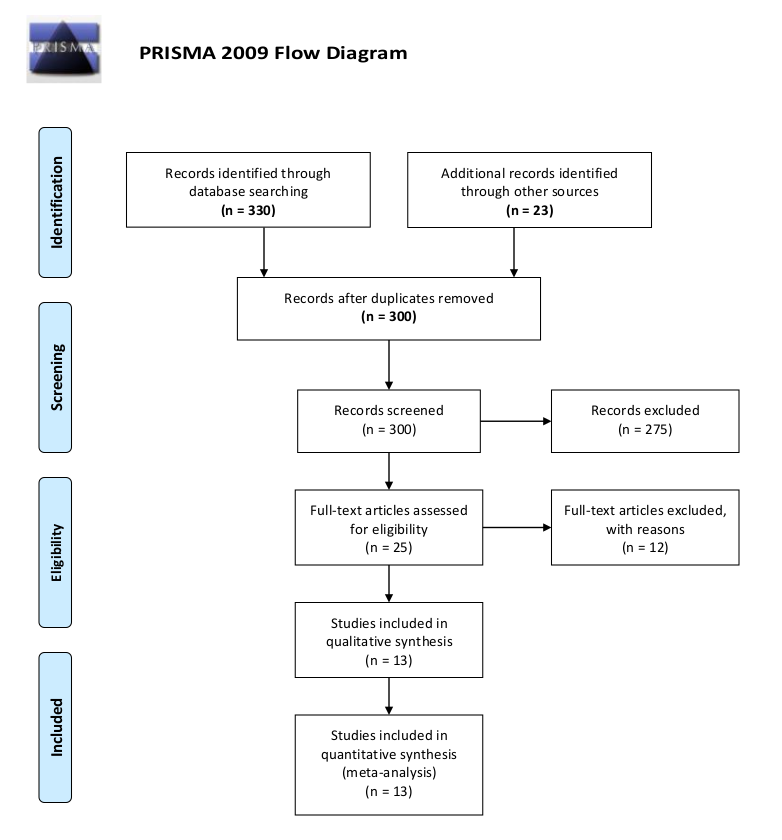

Supplement: Supplementary file 3 — Supplementary file3 (TIF 2680 KB) [file 384_2021_3963_MOESM3_ESM.tif]
